# Micro-coil probes for magnetic intracortical neural stimulation: Trade-offs in materials and design

**DOI:** 10.1063/5.0023486

**Published:** 2021-01-01

**Authors:** Krishnan Thyagarajan, Rene A. Lujan, Qian Wang, JengPing Lu, Sivkheng Kor, Bruce Kakimoto, Norine Chang, Julie A. Bert

**Affiliations:** Palo Alto Research Center (PARC), a Xerox Company, Palo Alto, California 94304, USA

## Abstract

Neural probes for intracortical neuromodulation in the brain have advanced with the developments in micro- and nanofabrication technologies. Most of these technologies for the intracortical stimulation have relied on the direct electrical stimulation via electrodes or arrays of electrodes. Generating electric fields using time-varying magnetic fields is a more recent neuromodulation technique that has proven to be more specifically effective for the intracortical stimulation. Additionally, current-actuated coils require no conductive contact with tissues and enable precise tailoring of magnetic fields, which are unaffected by the non-magnetic nature of the biological tissue and encapsulation layers. The material and design parameter space for such micro-coil fabrication can be optimized and tailored to deliver the ideal performance depending on the parameters needed for operation. In this work, we review the key requirements for implantable microcoils including the probe structure and material properties and discuss their characteristics and related challenges for the applications in intracortical neuromodulation.

## I. INTRODUCTION

Intracortical neuromodulation utilizing implantable neural probes has developed into a *de facto* standard in a number of clinical applications to treat a wide range of neurological (e.g., Parkinson’s disease^1^) and psychological (e.g., depression^2^) health issues. Soft implantable neural prostheses for this category of neuromodulation have also improved over the past decade, assisting the restoration and rehabilitation of patients suffering from paralysis.^3^ The long-term efficacy of electrode-based implants for intracortical stimulation is limited, however, with a few challenges that include an inability to create precise patterns of neural activity and difficulty maintaining a consistent response over time.^4,5^ The imprecise patterns generated by conventional electrodes result in the inadvertent activation of axons of distant neurons, reducing the acuity that is desirable in an implantable probe. This becomes especially important when specific locations of the intracortical regions need customized modulation. Variations in neural response are exacerbated by the inability to isolate the electrodes from the tissue, causing both tissue damage and probe deterioration over time. In the recent past, we have demonstrated several novel results using a novel form of the intracortical stimulation, namely, magnetic stimulation via the use of microcoils. In particular, in an earlier work, we described the first generation of the micro-coil design and demonstrated its effectiveness in both activating cortical neurons and driving behavioral responses^6^ *in vivo*. This work was performed both *in vitro* and *in vivo* in mice. Those findings suggested that a coil-based implant might be a useful alternative to the existing electrode-based devices. The enhanced selectivity of the micro-coil based magnetic stimulation may permit the special use for visual prostheses and potential brain–computer interface applications that require a precise activation of the cortex. Subsequently, we also demonstrated the effective use of a new generation of advanced microcoils that we developed at Palo Alto research center (PARC) to customize and individualize design features resulting in controlled influence over both the selectivity and strength of the neuromodulation^7^ to induce a desired response *in vitro*. The results showed how the coil design was able to influence the response of cortical neurons to stimulate extremely localized regions, with potential use as a part of future cortical prostheses. The fabrication of these advanced devices has given us insight into the design parameter space including spatial resolution, temporal resolution, *ex vivo* stability, and selectivity with which signals can be delivered, with the aim of assisting in the development of the next-generation cortical prostheses. Refer to the above-cited works, where details and insight into the functioning of the devices are discussed and details of the experimental findings are explained.

The micro-coil technology, introduced in earlier work,^6^ is a novel platform to enable the neural stimulation that is capable of overcoming the above limitations in the context of intracortical neuromodulation. In this work, we discuss the challenges and advantages that the micro-coil technology provides vis-à-vis the material properties and design properties of the microcoils themselves and offer design suggestions for intracortical neural stimulation devices. A comparison of the performance with current state-of-the-art electrode technologies for intracortical stimulation is also provided, and the advantages of our technology are discussed in detail.

This paper is organized as follows: Sec. II discusses the material properties that need to be considered when developing microcoils. In particular, it discusses the toxicity and biocompatibility (Sec. II A) of the substrate, metals (Sec. II A 1), and encapsulating layers (Sec. II A 2). We provide suggestions about which parameter values may result in desired outcomes of performance. Additionally, we explore optimizing the mechanical properties of the device to enable chronic implantation while reducing tissue damage and scarring, as discussed in the section on mechanical properties (Sec. II B). For the microcoils to be effective, they need to carry alternating currents, which generate time-varying magnetic fields. The metals used in the microcoils must have high conductivity to enable the use of smaller input voltages and enable smaller cross sections that reduce the physical impact of the insertion of the implants. The conductivity of the metals used in the microcoils also determines the heat generated (Sec. II C). An advantage of such contactless microcoils is that they allow the use of conventionally toxic metals with superior electrical properties to be encapsulated and isolated from tissue. After the material properties have been decided, there is still a large parameter space of micro-coil design (Sec. III) that can be optimized for the end application. In this regard, in this article, we discuss two important design parameters—the shape of the coils (Sec. III A) and the number of microcoils (Sec. III B). Finally, we conclude the work by summarizing all the above sections in Sec. IV. The micro-coil technology presented here has several distinct advantages over the conventional electrode technology for the use in intracortical stimulation and can thus provide a significant advantage for future advanced cortical prostheses.

## II. MATERIALS CONSIDERATIONS

Conventionally, electrodes and microelectrodes have allowed for safe and well-established means for implantation into the cortex. Most of the previous work thus far conducted with laboratory animals and in clinical testing has demonstrated the viability of intracortical neuromodulation via such an electrical stimulation. However, the efficacy and performance of such devices remain limited due to the inability to precisely target specific types of cortical neurons or even confine activation to specific cortical regions. Micro-coil probe technology solves a number of these challenges that conventional electrodes face for the intracortical stimulation.^6^ Table I highlights the advantages of the micro-coil technology in comparison to the state-of-the-art. There is a large design space within which we can choose the parameters for fabricating the probes. This includes (A) biocompatibility properties of metals/encapsulation layer, (B) mechanical properties, and (C) thermal properties. These properties are discussed in Secs. II A–II C.

**TABLE I.** Comparison of key attributes in state-of-the-art electrodes in comparison to our micro-coil technology (^6–8^).

Attribute	Intracortical electrodes	Micro-coils
Spatial resolution	50 *μ*m	60 *μ*m
Temporal resolution	0.1 ms	0.1 ms
Insulation	Parylene-C	Parylene-C, SiONx, SiO2
Resistance(*@*1 kHz)	50 kΩ–100 kΩ	10 Ω–40 Ω
Electrode length	0.5 mm–1.5 mm	1 mm–2mm
Electrode width	5 *μ*m	7 *μ*m–10 *μ*m
Metals used	Ir, Pt	Ag, Au, Cu, Pt

### A. Biocompatibility of metal and encapsulation material

The extent and kind of the immune response that is evoked by an implantable neural interface depend on a complex set of factors including, but not limited to, the mechanical force of insertion, the toxicity of the material, and the exposure of the tissue to external input signals (e.g., electrical currents).^9–11^ For the implantation of such devices, often surgical procedures are needed, which may damage target tissues, following which the body tries to restore tissue homeostasis in the form of wound healing. Chronic implantation may, further, overstimulate the immune system, leading to a complex chain of events involving the foreign body response. This may involve glial scar formation and the activation of macrophages, phagocytosis, and oxidative stress due to the presence of reactive ion species.^12,13^ Over time, such scar formation can create layers of insulation, thereby growing the distance between the intended stimulation site and the electrode itself, thereby degrading the performance of the implant.^14^ Among the various parameters to minimize this degradation, appropriate materials selection is important.

#### 1. Metal electrodes

**FIG. 1.**
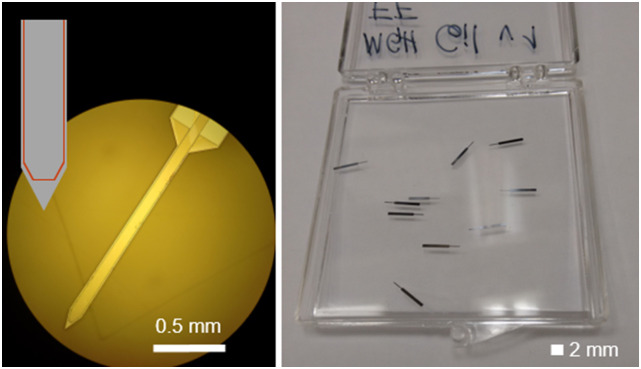
Optical microscope image of the copper coils (left) with the inset showing a sketch of the coil shape at the tip and a photo of the coils, showing the compactness of the design (right).

Metals are the most common material used for intracortical neural implants.^15,16^ Frequently used electrode materials include platinum and tungsten. These are considered biocompatible but can be expensive or of lower conductivity than a few other commonly available metals.^17,18^ In our experimental work with micro-coil probes, we have successfully used copper, silver, and gold as metals, keeping in mind economic costs as well. Silver and copper provide high electrical conductivity but are considered toxic as they tend to provoke a strong immune response.^19^ By using such metals in our micro-coil probes, we are able to access these high quality materials, which can drive down costs, since our devices do not have direct contact with the tissue. Our copper coils^6^ consisted of a copper trace (10 wide × 2 *μ*m^2^ thick) on a silicon substrate with a cross-sectional area of 50 × 100 *μ*m^2^ and a length of 2000 *μ*m (Fig. 1). The coil assembly had an average DC resistance of ∼20 Ω and was encapsulated with 300 nm of SiO_2_, which was deposited by plasma-enhanced chemical vapor deposition (PECVD). The impact of the insulation layer will be discussed in Subsection II A 2. However, it is pertinent to mention that by virtue of the magnetic nature of the stimulation, we can afford to use thick insulation layers and what are considered to be conventionally *toxic* metals. We were able to demonstrate activation of cortical neurons and the driving of behavioral responses. It was also demonstrated that the stimulation of cortical pyramidal neurons in brain slices was reliable and could be confined to small spatial regions (<60 *μ*m). Despite copper being considered a toxic metal for biological use, the magnetic nature of the stimulation allowed us to use thick encapsulation layers to protect the tissue and prevent toxicity related response *in vivo* due to the metal. This highlights how a microcoil-based implant can be a useful alternative to electrode-based devices.

**FIG. 2.**
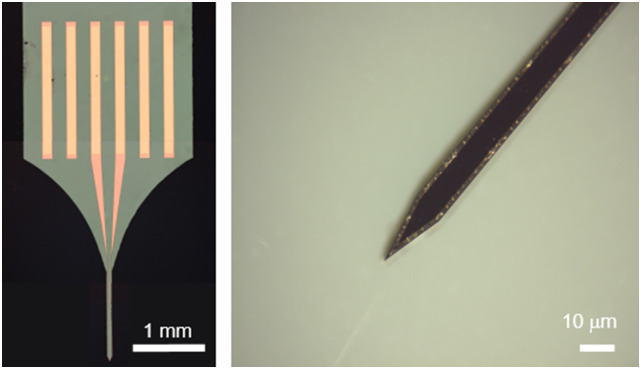
Optical microscope image of the gold coils (extreme left) and close-up view photo of the coil tip (right).

We also fabricated coils made of silver, which is considered less toxic than copper and provides high electrical conductivity. As shown in Table II, the average DC resistance of the coils (10 *μ*m width and 2 *μ*m height) is lower, ∼10 Ω–12 Ω, with a smaller standard deviation. *In vitro*, the coils achieved threshold activation of the neurons at lower input voltages when compared to the copper coils (used to generate the time-varying magnetic fields). We also experimented with gold microcoils,^7^ as gold does not have the toxicity concerns of silver or copper. We developed a fabrication technique that enabled the deposition of 2 *μ*m of gold as the conductive coil in a high-aspect ratio form factor. The average DC resistance of the coils was measured to be ∼30 Ω. We were able to deposit a greater thickness of gold, thereby reducing the need for larger voltages (Fig. 2). In conclusion, all three metals—gold, silver, and copper—delivered an optimal performance with no observed toxicity-related effects due to the presence of the insulation layer and the principle of neuromodulation with time-varying magnetic fields (permitting a thick insulation layer). One can thus take advantage of high conductivity of metals such as silver and copper despite their toxic nature when compared to gold and help drive down the price of commercial micro-coil based neural interfaces.

**TABLE II.** Summary of the useful properties of fabricated microcoils using different metals (toxicity and degradation are tabled without encapsulation).

Metal	Resistance (Ω)	Thickness (*μ*m)	Toxicity	Heating	Degradation
Copper	20 ± 3	2	Yes	No	Yes
Silver	12 ± 2	1	Yes	No	Yes
Gold	30 ± 5	2	No	No	No

Refer to our recently published work^7^ that discusses the details of the fabrication.

#### 2. Encapsulation layer

To avoid direct contact of the metals in the electrodes with the tissue, neural implants are often coated with an encapsulation or insulation layer.^20^ The most common materials used for this layer are dielectrics such as silicon nitride (SiN_*x*_), parylene-C, polyamide/polyurethane, and SiO_2_, which are deposited by either heat-shrinking, physical vapor deposition, dip coating, or electrodeposition.^14^ In addition to the biocompatibility of the material, it is important to ensure that the layer can be thin and maintain mechanical integrity, does not evolve volatile chemicals after implantation, and provides high-quality pinhole-free insulation.^21^ This last characteristic is important on multiple accounts: preventing the current passing through the coils from leaking into the surrounding tissue, preventing degradation of the metals via body fluids, and avoiding a toxicity-induced response from copper and silver microcoils. In our fabrication of the different microcoils, we have successfully used parylene-C, SiN_*x*_, and SiO_2_, depending upon the specific application and need for varying thickness of the layers. Although parylene-C is commonly used for flexible and corrosion-resistant medical devices such as electrodes and sensors, it can be challenging to integrate into different fabrication processes.^22^ This is aggravated by the poor adhesion at parylene–parylene and parylene–metal interfaces, which often cause probe failure after extended use.^23,24^

We observed that if the copper and silver microcoils have been exposed to air before the coating of the encapsulation layer, for thicknesses less than 100 nm, parylene-C is unable to form a pin-hole free layer that adheres well to the surface. We attribute this to the potential presence of adventitious carbon and formation of oxide layers on the metal surfaces. Furthermore, the presence of any excess moisture or sulfur on the surface seeds the formation of insulation layers before the encapsulation can be coated, leading to poor encapsulation and potential for toxicity. However, these limitations do not impact thicker layers (>2 *μ*m), nor do they affect the performance, if we use different encapsulation layers such as SiN_*x*_ and SiO_2_.

When using SiO_2_ as the insulation, due to the better control over the deposition parameters, we were able to use much thinner layers (∼200 nm–300 nm), thereby permitting a smaller cross section and less damage to the tissue when inserting *in vivo*. We observed that with optimized techniques of fabrication, we are able to obtain layers that remain well-insulated and functional even after exposure to air for several months and for extended periods of time *in vivo*. The pinhole-free nature of the deposition ensures the longer term stability of the microcoils even in saline environments. This has been tested out *in vivo*.^6,7^

### B. Mechanical properties of materials

For any neural interface to be effective for extended periods of implantation, it needs to minimize the foreign body response and glial scar formation, among other things. Among other factors, scarring is also dependent on the net thickness of the implant (in this case, the microcoils) and the encapsulation layer. The smaller the cross section, the less the damage to the tissue when the device is implanted. Previous studies have shown that when the electrode implant is comparable in size to neuronal bodies (∼12 *μ*m–15 *μ*m), less implantation trauma is suffered.^25^

Depending upon the intended location of operation, intracortical implants need to pierce through several non-homogeneous layers of tissue and potentially fibrous dura mater as well, before resting in the body. The insertion of the implant puts a large amount of force on the probe tip, requiring an appropriate design for distributing the pressure so as to not break the fragile tip. This becomes even more important with the greater adoption of CMOS-compatible materials for neural implants. Although the CMOS compatibility of many of the electrode technologies is a major advantage when trying to integrate electronics and optics with neural probes, the mechanical stiffness of these systems can be detrimental for neural implants, which need materials with lower Young’s Modulus.^15,16,26^ The brain’s Young’s modulus can range from around 1 kPa–10 kPa,^16,25,27^ while silicon has a value of 150 GPa–180 GPa. This makes excessive stiffness mismatch between the device and the brain tissue an issue. In addition, CMOS-compatible materials are not naturally biocompatible. The capability to use the encapsulation layers can potentially help bypass this issue.^28–35^

**FIG. 3.**
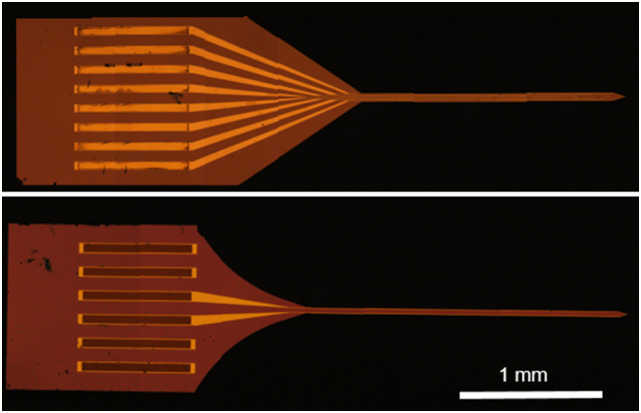
Optical microscope image of the difference in the design of the substrate shape, resulting in greater pressure (top) on the tip as opposed to the lower pressure on the tip (bottom) by the redistribution of the load due to the curved design of the substrate.

**FIG. 4.**
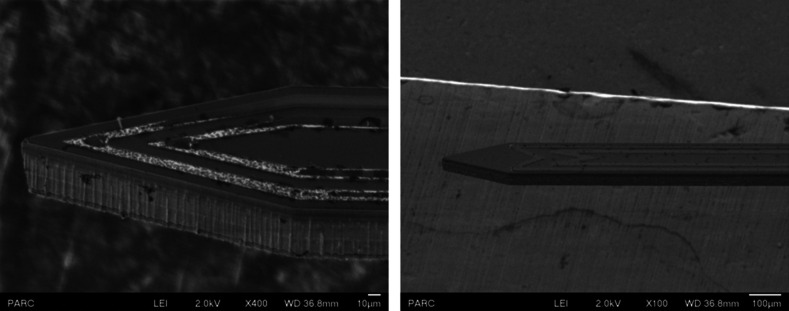
SEM microscope images of two different designs showing concentric V (left) and concentric W (right) designs showing the ability of the fabrication to create precise features (the dark dots on the device are due to the deposition of a conductive layer for SEM imaging).

Our probes consist of a 50 *μ*m thick silicon substrate on which the microcoils are designed. Our design enables distributing the pressure of insertion, significantly reducing both the breaking of the tips and insertion damage. Sharp corners are particularly susceptible for the substrate to crack, as there are strong mechanical pressure gradients along the intersecting lines. To address this, we designed curved edges joining the extended shank with the microcoils to the probe tip and the rest of contact pads (Fig. 3). This redistributes the pressure and reduces the gradient. Using curved tips, we observed a significant reduction in the broken tips upon insertion into tissue (up to ∼50%) and an average increase in lifetime (by up to ∼40%) when inserted. It is pertinent to mention that the back end of the devices is not embedded inside the tissue and therefore does not need a reduction in size. In the event that longer probes are needed, the length of the shanks can be increased and the back ends can be reduced in size. Scanning electron microscope (SEM) images (Fig. 4) of some of the fabricated microcoils show the precise interfaces that are formed. The dark dots on the devices in the images are due to the deposition of a conductive layer for the SEM imaging, since the device itself has an encapsulation layer that leads to charging of the device while imaging.

### C. Thermal properties

In operation, alternating currents are passed through the coils to generate time-varying magnetic fields, which, in turn, create the required electric field for neural stimulation. The coil resistance generates heat and the temperature rise must be limited to protect the living tissue. Higher electrical conductivity enables a smaller cross section to generate similar electric field strengths at the desired location. With the possibility of using metals with higher thermal conductivity (such as copper and silver) despite being toxic, we were able to ensure that the microcoils do not suffer a significant rise in temperature—as validated by monitoring the temperature rise in a bath and in the surrounding tissue.^6^

## III. COIL DESIGN

Once the materials have been selected, the performance of the microcoils can be further optimized through the coil design. Key parameters include the shape of the coils (IIIA) and the number of coils (IIIB). We discuss both of them individually in Subsections III A and III B.

### A. Electromagnetic field concentration by sharp interfaces

It is a well-known fact that sharp corners aid in what is known as the “lightning-rod effect,” the concentration of electric field lines at sharp points (“hot-spots”). Microcoils can be designed to have either single sharp points, for example, the tip of a V-shape, or multiple sharp points, for example, the tips of the W-shape.

**FIG. 5.**
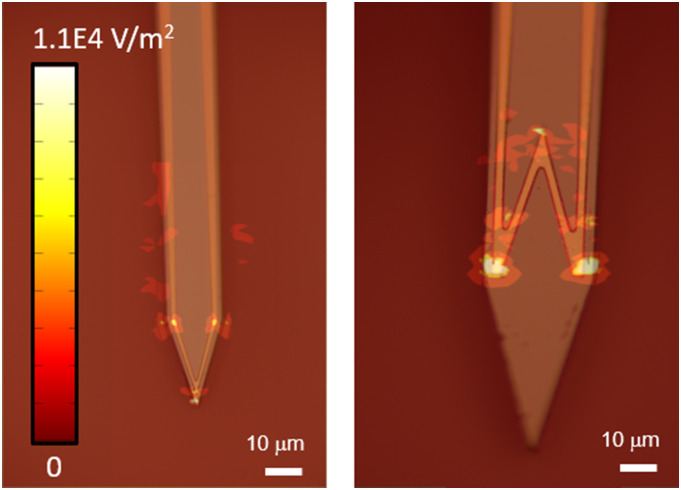
Optical microscope image of the gold single V-coil (left) and the single W-coil (right). Superimposed numerical simulations show that the strong electric field gradients for the V-shape (left) occur at fewer points than the W-shape (right) due to fewer sharp corners.

Numerical simulations carried out in COMSOL Multiphysics for a gold microcoil (Fig. 5) show greater electric field-gradients at multiple sharp corners for the W-shape when compared to the V-shape for a current of 1 mA at 5 kHz.^7^ Thus, for multiple points of activation, it is useful to have multiple sharp corners in the coil design. As was demonstrated in a recent study, the W-design performs better at selective excitation of multiple points.^7^ For multiple excitation points, it is conceivable to also design a sawtooth-like coil that enables selective excitation at pre-determined depths along the shank. This would be useful for simultaneously exciting pyramidal neurons in several layers of the cortex and could enable the study of the dynamics of intracortical activity.

### B. Electromagnetic field augmentation by concentric interfaces

**FIG. 6.**
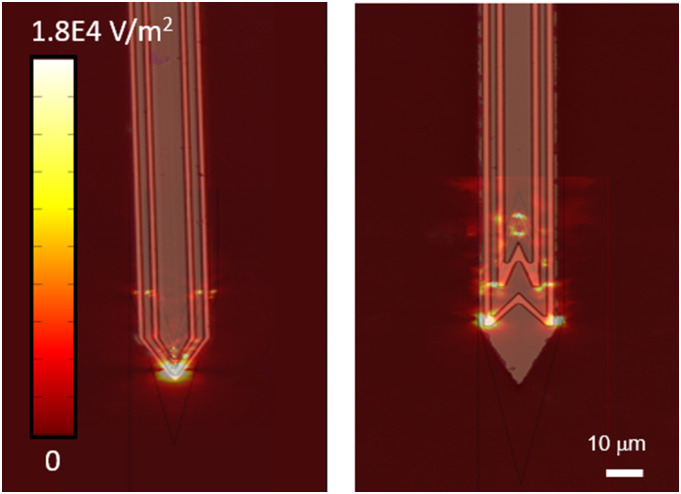
Optical microscope image of the gold concentric V-coil (left) and the concentric W-coil (right). Superimposed numerical simulations show that the strong electric field gradients for the V-shape (left) are stronger and cover a larger area than the W-shape (right).

To increase the electric-field gradients at a desired location, it is possible to design concentric microcoils that utilize multiple loops enclosing each other. In addition to the increase in the gradients, this also provides a depth-distribution of the hot-spots that can be used to simultaneously access different locations within the layers of the cortex. Figure 6 shows numerical simulations of two concentric V-shaped and W-shaped microcoils with the same operating parameters used for the single micro-coil simulations above and superimposed on their optical microscope images. The maximum value of the electric-field gradient increases when compared to the single coil analogues. In addition, while the W-shaped concentric coils provide localized excitation, the effectiveness of the stimulation in the concentric V-shaped coils is higher and can be used to excite neurons that are further away from the implant.

**FIG. 7.**
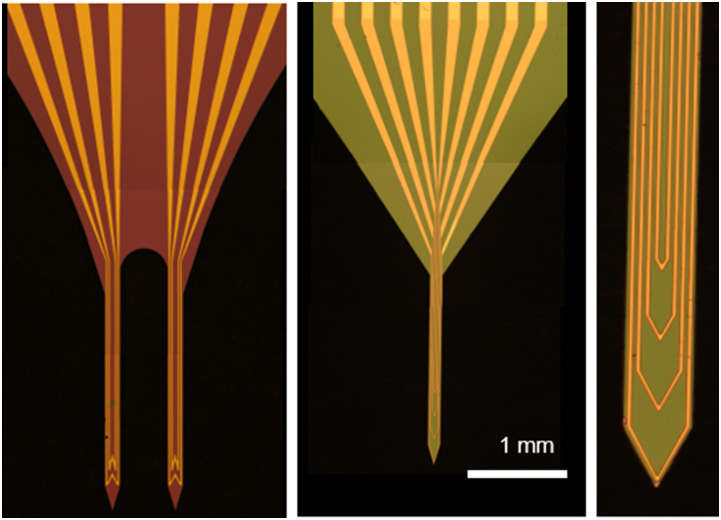
Optical microscope images of a double-shank double concentric W-shaped micro-coil design (left), quadruple concentric V-shaped micro-coil design (middle), and a close-up of the multiple concentric V-shaped microcoils.

Greater numbers of concentric coils permit greater control over the depth profile of the neural stimulation and the ability to excite different combination of layers in the cortex to study their influence on each other. We have also successfully fabricated and tested multiple adjacent shanks in the implant to simultaneously excite regions separated by more than a millimeter. Figure 7 shows examples of such implementations.

## IV. CONCLUSIONS

The use of magnetic stimulation via microcoils for intracortical neuromodulation has recently shown to be an effective alternative means to achieve highly localized neural activity. As evidenced by the recently published work, the biological performance of these devices has been shown to be comparable to microelectrodes and better than them for certain properties. These include lower resistance, greater variety of metal choices, and better chronic implantation for intracortical stimulation. In this article, we have highlighted the various advantages of using microcoils for the intracortical neural stimulation, along with the various parameters that need to be optimized depending on choice of operation. For any implantable intracortical neural interface to be successful and effective, it should enable precise neural stimulation above threshold while being biocompatible and stable to chronic implantation. Micro-coil technology offers several advantages over electrode-based stimulation for intracortical modulation, including the possibility to use materials with higher electrical conductivity without exposing the tissue to their toxicity and reduced impact of tissue scarring on the efficacy of the stimulation. The properties of the materials including the choice of metal, the encapsulation layer, and the choice of micro-coil design influence the final coil performance. In summary, the micro-coil platform offers a unique modality for intracortical stimulation, and the large parameter space of materials and design will enable greater adoption for intra-cortical neural stimulation in applications such as cortical prostheses.

## DATA AVAILABILITY

The data that support the findings of this study are available from the corresponding author upon reasonable request.
